# Exploring the relationship between audio–visual perception in Fuzhou universities and college students' attention restoration quality using machine learning

**DOI:** 10.3389/fpsyg.2025.1572426

**Published:** 2025-07-04

**Authors:** Shaofeng Chen, Zhengyan Chen, Jiawen Hong, Xiaowen Zhuang, Chenxi Su, Zheng Ding

**Affiliations:** ^1^College of Landscape Architecture and Art, Fujian Agriculture and Forestry University, Fuzhou, China; ^2^College of Foreign Languages, Fuzhou University, Fuzhou, Fujian, China

**Keywords:** Fuzhou, healthy campus, attention recovery, spatial perception, machine learning

## Abstract

**Objective:**

In response to the challenges posed by mental health issues among college students and the declining quality of campus environments, this study aims to reveal the complex mechanisms underlying the relationship between campus audiovisual environments and the quality of students' attention recovery. It further explores campus landscape optimization pathways driven by multi-source data, providing scientific basis for sustainable campus planning.

**Methods:**

Taking Fuzhou University Town as a case study, this study integrates machine learning technology with multi-source data (street view images, social media text, and PRS-11 questionnaires) to construct a “multi-modal perception mechanism analysis-dynamic evaluation iteration” framework. The CNN-BiLSTM model was used to predict attention recovery quality, combined with HRNet semantic segmentation, GBRT soundscape prediction, and CSV-T4SA sentiment analysis models to quantify audiovisual elements. XGBoost models and SHAP interpretability analysis were employed to reveal the effects and interaction mechanisms of variables.

**Results:**

(1) Attention recovery quality is significantly higher in liberal arts and agricultural/forestry universities than in science and engineering universities, with boundary effects and the synergistic design of humanistic soundscapes being key factors; (2) SHAP analysis identifies humanistic soundscapes, natural soundscapes, and color complexity as core influencing factors, with their effects exhibiting significant threshold characteristics; (3) Linear interaction mechanisms among audiovisual elements are discovered, such as the interaction between vegetation density and building enclosure degree enhancing recovery efficacy, and the synergistic design of musical soundscapes and paving materials can optimize perceptual experiences.

**Conclusion:**

By innovatively integrating multi-source data and machine learning techniques, this study systematically analyzes the relationship between campus audiovisual environments and attention recovery, breaking through the limitations of traditional linear analysis. The proposed “threshold response design” and “cross-modal collaborative optimization” strategies provide a new paradigm for campus planning, validate the scientific value of multi-sensory interaction design for mental health promotion, and offer a transferable methodological framework for global university environmental upgrades.

## 1 Introduction

### 1.1 The real background of mental health crises among college students

Reports from colleges and universities around the world say that college students are having various mental health problems (Karyotaki et al., 2020). A report from 2022 on the mental health of Chinese college students says that almost 80,000 students in China, aged 15–26, feel like their lives are full of doubt and instability. They face multiple pressures, including shifts in their lifestyles and learning methods and intense competition for higher education and jobs, along with various other challenges (Fu et al., 2023). The four dimensions of Attention Restoration Theory (ART)—being away, extent, fascination, and compatibility—have been shown to help students' cognitive fatigue (Ma et al., 2023). Natural elements on campus, such as soundscapes and green areas, can make this happen. The quality of the campus setting is facing two problems at the same time, which makes this figure even more important as Chinese universities continue to grow. First, the amount of green space built into new buildings has gone down from 35% in 2010 to 28% in 2020 (www.pishu.com.cn). Second, the amount of road noise on campus has gotten 6.2 decibels louder since last year (Wang et al., 2010). As of 2018, China had 2,663 general higher education institutions. These schools are home to 38.33 million college students who live, study, and do research on sites across the country. Based on this size, China is the world's largest provider of higher education. Cuijpers et al. (2019) did a study of college students and found that most of them were bored with the mental health services their schools offered and felt uneasy about asking psychological questions. Campuses of universities are not only where students do most of their daily activities, but they are also excellent places for organized social interaction, learning, and communication, and they have a lot of potential to improve mental health. As a result of making improving students' mental health a national priority, it is now more important than ever to give them effective ways to deal with mental tiredness.

### 1.2 Environmental health theory and current research

High-quality campus landscapes are an important part of ecological city construction and campus habitat. They not only meet the basic needs for ecological, aesthetic, and public activities but also play a crucial role in supporting the environment necessary for college students to study and live, thereby aiding in the recovery of student attention (Malekinezhad et al., 2020). Previous research indicates that greater perceived greenness is associated with an improved quality of life among students. Additionally, the restorative characteristics of certain campuses are shown to be significantly influenced by mediating factors (Hipp et al., 2016). Natural environments in campus spaces can foster enjoyable experiences, enhancing students' engagement in learning and participation in campus activities (Hipp et al., 2016; Hajrasouliha, 2017). These settings also support emotional regulation and contribute to maintaining mental health (Kexin et al., 2024). However, extant research frequently focuses on the restorative attributes of specific campus areas and primarily examines public space design, including green spaces and waterfront areas. In order to comprehensively understand the restorative potential of campus environments for student mental well-being, we draw upon three complementary theoretical frameworks. Attention Restoration Theory (ART) (Kaplan, 1995) provides a cognitive perspective, positing that exposure to natural settings can mitigate attention fatigue and enhance cognitive function through four key mechanisms: being away, extent, fascination, and compatibility. Stress Reduction Theory (SRT) (Ulrich, 1983) offers a psychophysiological perspective, suggesting that natural environments rapidly reduce psychological distress and negative affect by eliciting positive emotional responses and reducing physiological arousal. The Biophilia Hypothesis (Kellert and Wilson, 1995) provides an evolutionary perspective, proposing an innate human affinity for nature, where natural elements inherently attract attention and foster positive psychological states. Critically, attention restoration (a core outcome of ART) serves as a measurable indicator of the cognitive recovery process, which is intrinsically linked to broader mental health outcomes targeted by SRT and underpinned by the biophilic connection. For instance, sustained attention fatigue is a recognized precursor to stress and diminished well-being, while restored attention facilitates coping and engagement. Therefore, assessing attention recovery quality provides a focused and empirically tractable lens through which to evaluate the broader restorative benefits (encompassing stress reduction and biophilic fulfillment) of campus audiovisual environments on student mental health. However, despite the synergy between these theories, extant research has been deficient in conducting a comprehensive, multidimensional analysis of the restorative attributes of campus environments, particularly integrating both auditory and visual perception within this combined theoretical framework (Lu and Fu, 2019).

### 1.3 Current status of audio–visual interaction research

While many studies acknowledge that perceptual restoration in humans arises from both visual and auditory stimuli, current research has primarily concentrated on visual perception (Liu et al., 2018; Yilmaz et al., 2023). Soundscape, as defined by the International Organization for Standardization (ISO), refers to the acoustic environment as experienced by individuals within a specific context. This definition highlights its critical importance in interpreting the surrounding environment, which plays a fundamental role in promoting public health and overall well-being. For example, White et al. (2010) demonstrated that natural sounds have a significant impact on restoration outcomes. In addition, human-generated noise, such as traffic noise, sounds of mechanical activities, and human chatter, for example, adversely affects public health. Li et al. (2024) explored how three different spatial configurations of water sound influenced the perception of traffic noise, employing a portable electroencephalogram to capture participants' brain activity. Although existing research has revealed the synergistic effects of audiovisual interaction on human perception recovery (Lindquist et al., 2016; Chen L. et al., 2024), currently, research on the interaction between audiovisual interaction and health benefits has mainly focused on, for example, how urban park soundscapes promote psychological and physiological recovery in children to a certain extent (Shu and Ma, 2020), the objective is to identify the structural characteristics of urban forest sound sources and to explore the differences in soundscape characteristics of landscape spaces and their influencing factors (Zhao et al., 2022), Audio information influences perceptions of the naturalness of urban landscapes (Jeon and Jo, 2020), and understanding how audiovisual interaction specifically influences students' perceptual recovery and health benefits in university green spaces (Ma et al., 2023). The current research paradigm still faces three key limitations: First, in terms of research objects, most studies focus on natural-dominated environments such as urban parks and forests (Shu and Ma, 2020; Zhao et al., 2022), while neglecting university campus environments with unique cultural attributes and functional zones. Different college buildings, historical areas, and teaching spaces combine sounds and visuals in very distinct ways, and we haven't yet fully analyzed how people perceive these combinations in this educational setting. Second, due to the lack of efficient research methods for large-scale acquisition of audiovisual elements and soundscape features, these studies have certain limitations in terms of depth and breadth. This situation further highlights the necessity of deepening research on the relationship between audiovisual interaction and environmental restoration in different universities and their campus cultural contexts to comprehensively enhance our understanding of their interactive mechanisms and their impacts in diverse campus and cultural landscapes. We have comprehended the precise impact of audiovisual interaction on the restoration of students' perception and the health advantages in university green spaces.

To effectively assess audiovisual impacts in different environments, the high cost and small scale of current methods have been addressed to some extent. However, the difficulty of controlling the quality of these data suggests the need to investigate new data sources for sound level assessment (Hsieh et al., 2015; Verma et al., 2019; Gasco et al., 2020). In the context of research methodologies and framework development for understanding its influence mechanisms, advancements in machine learning techniques and the proliferation of social media platforms have opened new avenues for studying Attention Recovery Perception among college students. These innovations significantly enhance the efficiency of data collection and the analysis of complex metrics. On this basis, many studies have analyzed the coupled relationship between the environment and human emotions using machine learning methods and user-generated content (UGC), such as the GBDT model (Ma et al., 2023), XGBoost (Chen Z. et al., 2024), and Word2vec (Zhang et al., 2023). The current standardized evaluation system using big data is yet to be further developed, for example, there are difficulties in how to adopt a flexible and dynamic strategy on data collection, processing and model training in the rapidly changing environment of social media. Therefore, there is an urgent need to develop an urban audiovisual prediction and assessment framework that can be accurately assessed, low-cost, large-scale and high-resolution, enabling design practitioners and managers to better utilize the big data environmental assessment model, thus improving the efficiency of university college renewal planning and further enhancing the ease of use of contemporary experimental methods and the practicability of experimental results.

### 1.4 Research objectives

To address the lack of research on the relationship between audiovisual perception and attention recovery on university campuses, this study selected a university town in Fuzhou, China, as its research site. Utilizing machine learning technology, the study focused on two key research questions: first, to reveal how audiovisual perception in campus environments influences the quality of attention recovery among university students through complex mechanisms; second, to conduct an in-depth analysis of the dynamic impact of audiovisual environments on this recovery process. Current related studies lack structured frameworks and interdisciplinary evaluation systems, and they overlook the interactive effects between audiovisual perception and attention recovery. Based on this, this study sets the following objectives: (1) to explore the complex mechanisms through which campus audiovisual perception influences attention recovery, adapting to changes in the digital environment through data collection and model training; (2) to construct an interdisciplinary evaluation framework, integrating machine learning methods to study the relationship between audiovisual perception and attention recovery, and providing data support for related research; and (3) to propose sustainable planning recommendations, optimizing the design of Fuzhou University campus based on research findings, and supplying reference for global campus planning.

## 2 Methodology and data

### 2.1 Research area

**Figure 1:**
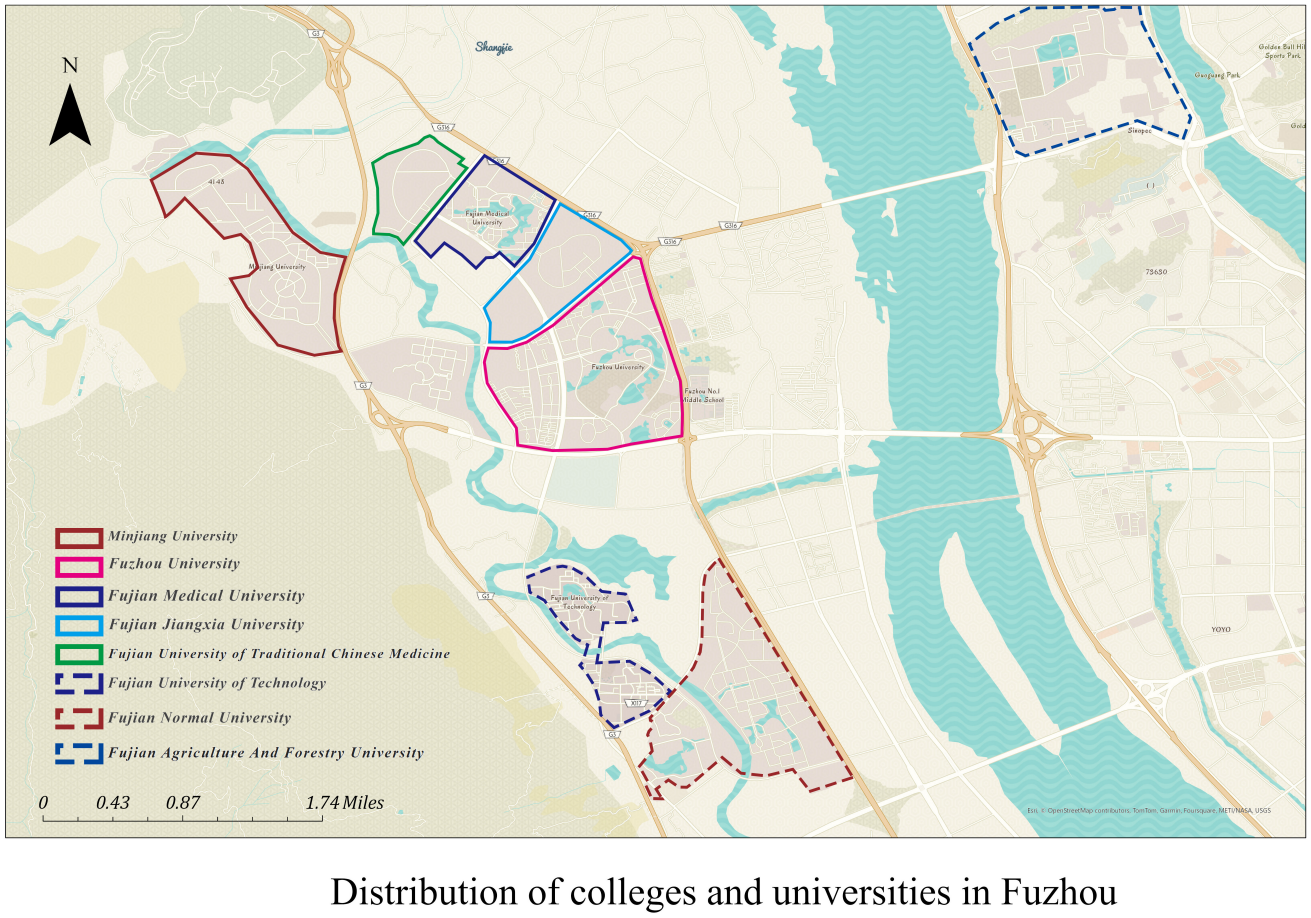
Study area.

Fuzhou, the capital of Fujian Province in China, is home to Fuzhou University Town, located in Shangjie Town, Minhou County. This multi-functional park seamlessly integrates education, culture, ecology, and daily life. Currently there are 13 colleges and universities, including Fuzhou University, Minjiang College, Fujian University of Technology, Fujian Medical University, Fujian University of Traditional Chinese Medicine, Fujian Jiangxia University, etc. This development not only drives growth in the Shangjie area but also creates a significant clustering effect in Jinshan New District. In terms of location choice, Fuzhou University Town is located in the strategic direction of the city's westward development, with the Qishan Mountain Range to the west and the Wulong River to the east, providing a superior natural ecological environment. In addition, the study area joins the Jinshan Campus of Fujian Agriculture and Forestry University, which is known as an “ecological garden university”. Guanyin Lake is the largest artificial lake in the campus, which is also an important channel for teachers and students to experience the natural landscape and ecological culture, and it can provide a control experimental group. Therefore, the study area includes Fuzhou University, Fujian Normal University, Minjiang College, Fujian Medical University, Fujian Jiangxia University, Fujian University of Traditional Chinese Medicine, and Jinshan Campus of Fujian Agriculture and Forestry University. Figure 1 shows the extent of the study area.

### 2.2 Conceptual framework

**Figure 2:**
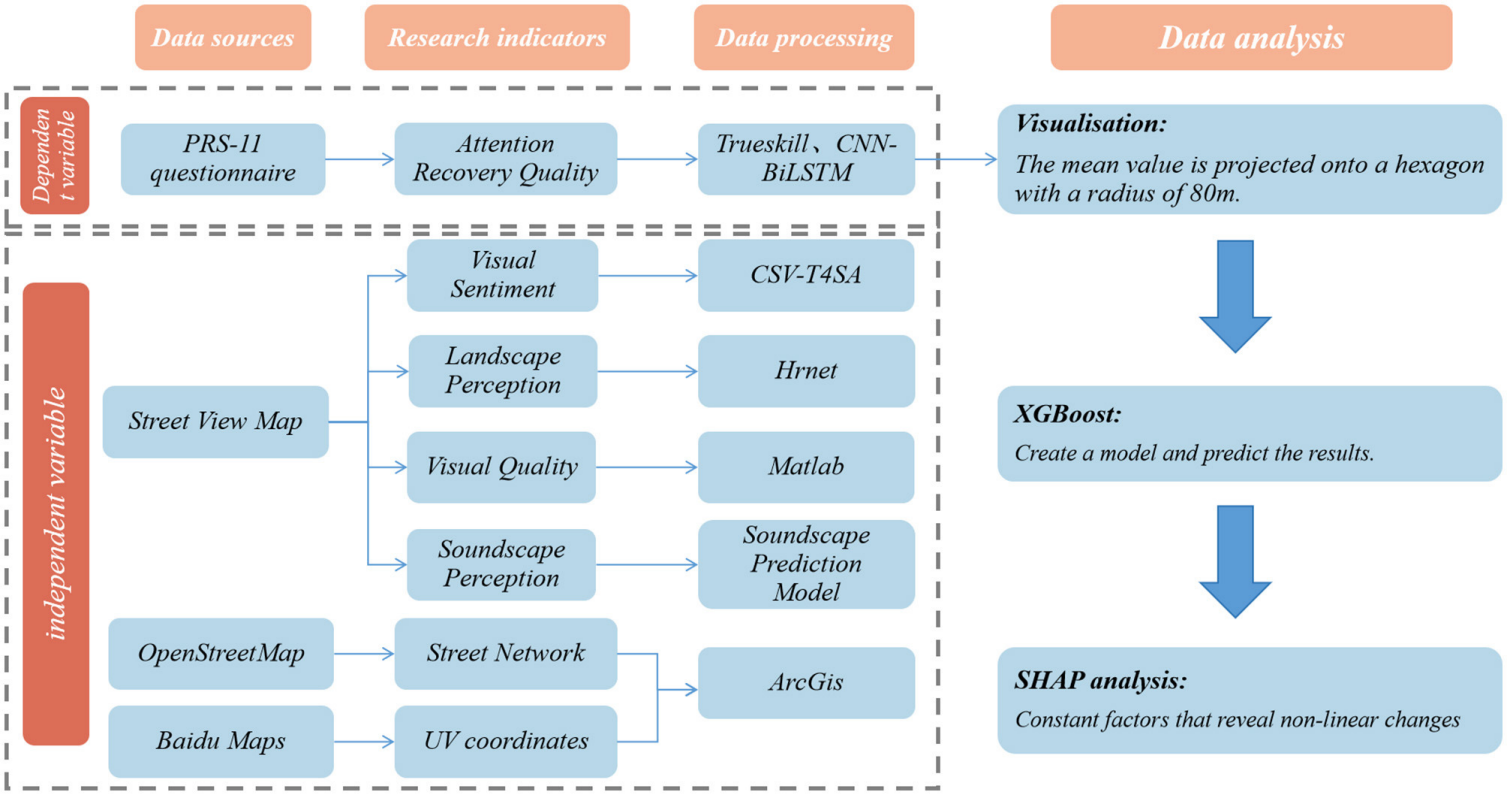
Theoretical framework.

To address the triple limitations of extant studies in campus audiovisual restorative assessment—i.e., unimodal perception limitation, linear analysis limitation, and static data limitation—the present study integrates PRS-11, SVI, CSV, T4SA, soundscape prediction, and geoanalysis methods through the innovative framework of “multi-source perception fusion—non-linear mechanism analysis—dynamic assessment iteration.” The integration of T4SA, soundscape prediction, and geoanalysis methods (Figure 2) has been shown to produce a synergistic effect. The experiment integrates the unique strengths of various machine learning methods and proposes a system that can be regularly run offline or online by continuously learning from streaming social media data. The “Cross-modal Distillation Paradigm for Constructing Sentiment Perception (CSV-T4SA)” was combined with Trueskill Matching Perception computation (Allouche et al., 2006). The experiment was followed by the utilization of Hrenet and Matlab high-resolution and compatible processing to ensure the quantification of landscape image quality. Concurrently, computer vision is employed to extract the visual features of SVI at four levels: pixel-level features, object-level features, semantic-level features, and scene-level features. These features are then utilized to construct a soundscape prediction model, in which the SVI features and soundscape labels are employed as inputs to train a Gradient Boosted Regression Tree (GBRT) model. The resultant soundscape of cities can be mapped by feeding city-scale SVI features into the learning model (Zhao et al., 2023). Finally, ArcMap software verifies the autocorrelation between different variables and spaces through its spatial autocorrelation analysis and hotspot analysis, which ensures the visibility and readability of the results. Finally, the XGBoost and SHAP algorithms were used to thoroughly examine the reasons behind the changes in the quality of attention restoration.

### 2.3 Variable selection

To ensure objectivity in selecting experimental metrics and facilitate meaningful comparison with peer studies, we conducted a review of key publications on the topic from the past decade. From this, we identified quantifiable metrics of attentional restorative perception (ART) used in relevant studies and enhanced them with experimental innovations.

Among existing studies, Attention Restoration Theory identifies four key factors for evaluating restorative environments: Being-away, Extent, Fascination, and Compatibility. These factors provide valuable conceptual frameworks for understanding restorative perceptions of physical environments. The notion of Being-away, for example, allows individuals to mentally detach from the stressful environments frequently encountered in daily life (Kaplan, 1984). Lee et al. (2015) further demonstrated that briefly observing the outside world during a break can facilitate mental relaxation. Extent aids in cognitive recovery by fostering a sense of engagement and immersion. Certain studies have classified this concept into coherence and scope. Coherence describes the systematic alignment and interrelation of elements within a scene, whereas scope highlights the possibilities offered by the environment for discovery and participation, enhancing the perception of spatial profundity (Hartig et al., 1997). Each factor aids in the development of a cognitive environmental framework that promotes active participation and deep focus. Kaplan (1995) defines fascination as an innate attraction to the environment, allowing individuals to engage without draining their attentional capacity or requiring the recovery of depleted cognitive resources. Compatibility is characterized by the harmony between an individual's goals and the activities facilitated by the environment. It relies on the individual's capacity to adjust to their surroundings and remains adaptable over time (Celikors and Wells, 2022). As this research emphasizes the visual attributes of the environment, factors associated with compatibility were excluded from the variable selection process. Tree canopy density and views of greenery through windows have been shown to exhibit a strong positive relationship with the well-being, life satisfaction, and academic success of college students (Markevych et al., 2017). Paths with level surfaces and hard sidewalks attract students engaged in dynamic sports such as walking, running, and biking (Holt et al., 2019). However, certain students prefer water-rich areas for exercise (Massoni et al., 2018). Additionally, the openness of the scene and the presence of artificial amenities can also influence individuals' emotional responses (Kotabe et al., 2017). And it has been mentioned in the introduction that the sounds of nature, traffic, mechanical activities, and human chatter have some impact on people's physical and mental health.

First, we adopted the PRS-11 scale proposed by Laumann to assess the “Being-away,” “Coherence,” “scope,” and “Fascination” in campus restoration effects (Laumann et al., 2001). To establish the experimental ecological validity of attention recovery research, we introduced a stress-inducing phase prior to the scale assessment: participants were required to complete a 30-s positive mental arithmetic stress test (two-digit multiplication), simulating real-life stressors such as academic pressure to establish a baseline state of individual attention resource depletion. This design enables subsequent PRS-11 scale scores to effectively capture the promotional effects of environmental characteristics on post-stress cognitive recovery, aligning with the methodological framework of attention recovery theory. Unlike traditional PRS scales, the PRS-11 includes two indicators describing scope and three indicators describing coherence and appeal. Drawing on Celikors' research (Celikors and Wells, 2022), we selected the most descriptive phrases for each quality and asked participants to rate their agreement with the image descriptions on a scale from 1 (strongly disagree) to 7 (strongly agree) (Table 1). This operational procedure ensures that the scale measures immediate environmental perception responses following stress exposure, rather than routine judgments under steady-state conditions. The final questionnaire achieved a Cronbach's alpha coefficient of 0.815 (Table 1), primarily capturing the cognitive restorative dimensions outlined by ART (Being-away, Coherence, Scope, Fascination). To enhance ecological validity and align with the stress-inducing contexts relevant to SRT, participants performed a 30-s active mental arithmetic stress test prior to the questionnaire, simulating real-life academic pressures to establish a baseline state of attention resource depletion. Secondly, acknowledging the notion that positive emotional responses (a fundamental mechanism in SRT and an outcome associated with biophilia) can mediate or co-occur with attention restoration (Ma et al., 2023). For this reason, the assessment of “college students' positive emotions” in this experiment utilized the CSV-T4SA cross-modal distillation paradigm, which was specifically pre-trained with multimodal (social platform text combined with image data) to construct a model of sentiment perception. In conclusion, to achieve a more accurate assessment of the visual experiences of university students, we integrated visual analysis metrics, including Visual Entropy and Color Complexity, which were analyzed using Matlab software. These metrics were used to assess the visual quality of the landscape configuration (Table 2).

**Table 1:** PRS-11 scale.

**Restorative quality**	**Descriptions**	**Score (7-pointLikert scale)**
Being-away	“To stop thinking about the things that *I* mustget done *I* like to go to places like this.”	poor 1–7 good
Coherence	“It is easy to see how things are organized in this place.”	poor 1–7 good
Scope	“This place is large enough to allow exploration in many directions.”	poor 1–7 good
Fascination	“In this place, my attention is drawn to many interesting things.”	poor 1–7 good

**Table 2:** Research element breakdown table.

**Research components**	**Research metrics**	**Metrics description**	**Quantitative approaches**
Dependent variable	Attention restoration quality	Attention restorative quality score in campus landscape	Trueskill/CNN-BiLSTM
Independent variables	Visual sentiment index	visual perception score in landscape perception	CSV-T4SA
	Color complexity	Color diversity in images	Matlab
	Visual entropy	Image entropy value	Matlab
Independent variables	Surface paving	The proportion of surface coverage in the image	HRNet
	Waterscape	The proportion of water feature in the image	HRNet
	Architectural enclosure	The proportion of architectural enclosure in the image	HRNet
	Vegetation density	The proportion of green plants in the image	HRNet
	Openness	The proportion of sky in the image	HRNet
Independent variables	Natural sound	The natural sound of streetscape image prediction	GBRT
	Humanistic sound	The humanistic sound of streetscape image prediction	GBRT
	Mechanical sound	The mechanical sound of streetscape image prediction	GBRT
	Musical sound	The musical sound of streetscape image prediction	GBRT
	Traffic sound	The traffic sound of streetscape image prediction	GBRT
	Noise intensity	The noise intensity of streetscape image prediction	GBRT
	Sound quality	The sound quality of streetscape image prediction	GBRT

Furthermore, objective visual (e.g., Vegetation Density, Waterscape proportion, Openness) and auditory metrics (e.g., Natural Sound proportion, Humanistic Sound proportion) derived from SVI analysis serve as quantifiable proxies for the presence of natural elements central to the Biophilia Hypothesis and the restorative processes described by both ART and SRT. The quantification of soundscape characteristics (e.g., Noise Intensity, Musical Sound) further allows us to examine environmental factors that may impede (SRT) or facilitate (ART, Biophilia) restoration.

### 2.4 Data acquisition and processing

#### 2.4.1. Baidu street view images (SVI)

In August of 2024, we obtained road network data of varying levels from the OpenStreetMap (OSM) of Fuzhou City. The “Create Grid” tool in ArcGIS 10.8 was utilized, with the OSM road network topology serving as the foundation. This approach yielded a set of evenly spaced 50-meter sampling points (Ogawa et al., 2024). The conversion of these points was executed through the utilization of a projection coordinate system, a method that ensures the precision of planar distances, with the road centerline serving as the reference point. To guarantee the geographical accessibility of the sampling points, Baidu Maps' route planning API verified that all points were situated on public roads. Subsequently, the Baidu Street View Static Map API (version v3) was employed to retrieve historical street view images corresponding to the sampling points. The images under consideration were collected by Baidu between June and October of 2021, 2022, and 2023. In order to ensure consistency with regard to lighting and vegetation conditions (such as leaf density), only images captured between 9:00 a.m. and 5:00 p.m. local time were extracted. This was done to minimize the impact of varying lighting conditions on image quality. Uniform parameters were established, and Baidu Street View Static Map API (version v3) was utilized for image crawling. The parameters encompass an image resolution of 1,600 × 1,200 pixels, a horizontal field of view of 90°, a vertical field of view of 30°, and a camera height of 2.5 m. This configuration was utilized to simulate a pedestrian's perspective. At each sampling point, four images were collected in the compass directions of 0° (north), 90° (east), 180° (south), and 270° (west). These images were then stitched into a 360° panoramic image using the OpenCV 4.5 stitching module. Consequently, the discrepancies in images and deviations in sampling are manually verified. Two processors independently assess the sampled images, removing blurry images and overly exposed images, and verifying the exclusion of non-traditional campus roads, such as highways and construction-closed roads.

#### 2.4.2. Cross-modal distillation paradigm for constructing emotion perception models (CSV-T4SA)

The CSV-T4SA model, constructed by Serra et al. (2023), is an automated sentiment polarity classification model based on a cross-modal distillation paradigm, specifically for visual sentiment analysis of social media images. The core of the model is to utilize multimodal (text + image) data to predict the sentiment polarity of images by using the output of a textual teacher model to guide the training of a student model on the visual modality. As user-generated content (UGC) data on social media continues to grow and change, the model can be adapted to new data distributions and features through continuous learning and updating. This allows the model to maintain its sophistication and accuracy, serving the field of social media sentiment analysis for a long time. When tested on five manually labeled benchmarks, the model outperforms the current state-of-the-art. This demonstrates the accuracy and reliability of the model in predicting image sentiment polarity.

#### 2.4.3. Perceiving soundscape models from street view images

The connection between soundscapes and human visual perception has been shown to be substantial, with visual characteristics derived using three separate pre-trained deep learning models. At the pixel level, features such as hue, saturation, luminance, and edge detection values were obtained using algorithms from the OpenCV library. For object-level feature extraction, the Faster R-CNN model (Ren et al., 2017), trained on the COCO dataset (Lin et al., 2014), was utilized to detect and count elements within 91 object categories, including pedestrians, buses, and traffic signals. Semantic-level feature extraction was conducted using the DeepLabV3+ model (Chen et al., 2018), trained on the Cityscape dataset (Cordts et al., 2016), which classifies over 19 categories, such as vegetation, sky, and architecture, from ground-level imagery. Additionally, to estimate scene attributes in SVI, the ResNet model (He et al., 2016), trained on the Places365 dataset (Zhou et al., 2015), was implemented. This dataset spans 365 scene types, including green parks, school buildings, gymnasiums, canteens, libraries, and other areas related to campuses. Leveraging SVI, this research investigates the interplay between visual attributes and human perception, aiming to pinpoint critical visual elements that elicit specific perceptual responses (Herzog et al., 1976).

In this study, the pre-trained gradient boosted regression tree (GBRT) model developed by Zhao et al. was used for campus soundscape prediction, and the specific process included three steps: First, pixel-level feature, object-level feature, Semantic-level feature, and overall scenes were gathered from the campus street scene data; second, the pre-trained GBRT model was used to predict where artificial sounds (like human voices) and natural sounds (like bird songs and running water) would occur on campus. Secondly, the pre-trained GBRT model is used to spatially predict artificial sound sources (e.g., human sound activities) and natural sound sources (e.g., bird songs, running water sounds) in the campus; finally, the model performance is evaluated by using the Mean Absolute Error (MAE = 0.152–0.372) and the Coefficient of Determination (*R*^2^ = 0.521–0.689), and the results show that the model can efficiently capture the spatial distribution patterns of different soundscape elements.

#### 2.4.4. High Resolution Network (HRNet) for semantic segmentation

The HRNet model, renowned for its robust generalization ability and consistent performance in analyzing landscape element data from images, is applied in this analysis. By integrating high- and low-resolution convolutions simultaneously, the model significantly improves the accuracy and efficiency of semantic segmentation tasks. HRNet achieves state-of-the-art results across various semantic segmentation benchmarks, including Cityscapes and PASCAL Context. On the Cityscapes dataset, in particular, HRNet outperforms other advanced segmentation models such as DeepLabV3+ and PSPNet, demonstrating exceptional proficiency in managing complex visual scenes (Sun et al., 2019). The model excels in handling intricate semantic segmentation tasks while maintaining relatively low computational requirements, making it well-suited for processing large-scale datasets, such as campus landscape images. During the experiments, HRNet effectively extracted critical metrics, including openness, water view ratio, green view proportion, paving consistency, and building enclosure levels, from the images. The methodologies for calculating these landscape element metrics are grounded in the work of Wu et al. (2023), with the detailed computational procedures for each indicator provided in Table 3.

**Table 3:** Explanation of semantic segmentation recognition.

**Research metrics**	**Calculation of metrics**
Surface paving (SP)	SP = (*P*_paving_ / *P*_total_) ^*^ 100%, where SP is the percentage of Surface paving, *P*_paving_ is the number of paving pixels, and *P*_total_ is the total number of pixels in the image.
Water feature proportion (*W*)	*W* = (*P*_water_/*P*_total_) ^*^ 100%, where w represents the water feature proportion in the image; *P*_water_is the total number of pixels identified as water elements by the model; *P*_total_ refers to the total number of pixels identified in the image.
Architectural enclosure (*A*)	*A* = (*P*_Enclosure_/P_total_) ^*^ 100%, where A is the percentage of the buildings and walls; *P*_Enclosure_ is the pixel count for architectural structures identified by the model; *P*_total_ is the total pixel count in the image.
Vegetation density (*V*)	*V* = (*P*_Vegetation_ / *P*_total_) ^*^ 100%, where V is the vegetation coverage percentage; *P*_Vegetation_ is the pixel count of green elements; *P*_total_ is the total pixel count.
Openness (*O*)	*O* = (*P*_Sky/_ *P*_total_) ^*^ 100%, where O is the openness value of the image; *P*_Sky_ is the number of pixels identified as sky elements; *P*_Total_ is the total pixel count in the image.

#### 2.4.5 Convolutional Neural Network—Bidirectional Long Short Term Memory (CNN-BiLSTM)

The rapid advancement of artificial intelligence has made deep learning a pivotal tool for handling complex datasets. Among the various models, Convolutional Neural Networks (CNNs) (LeCun et al., 1989) and Long Short-Term Memory (LSTM) networks (Hochreiter and Schmidhuber, 1997) are two of the most commonly employed deep learning architectures. With the swift progress of artificial intelligence, deep learning has become a crucial tool for processing complex datasets. Convolutional Neural Networks (CNNs) (LeCun et al., 1989) and Long Short-Term Memory (LSTM) networks (Hochreiter and Schmidhuber, 1997) are among the most widely adopted deep learning architectures in this context. Combining these two models to form the CNN-BiLSTM architecture can simultaneously.

By leveraging their respective strengths, a prediction model is achieved through the integration of Convolutional Neural Networks and Bidirectional Long Short-Term Memory Recurrent Neural Networks for classification tasks. Traditional machine learning models, such as SVM and Naive Bayes, depend on manual feature engineering, which limits their ability to effectively capture complex patterns and deep features within text. In contrast, the CNN-BiLSTM algorithm excels with higher recognition accuracy, a lower average leakage rate, and reduced latency.

#### 2.4.6 Image computing method based on matching mechanism (Trueskill)

**Figure 3:**
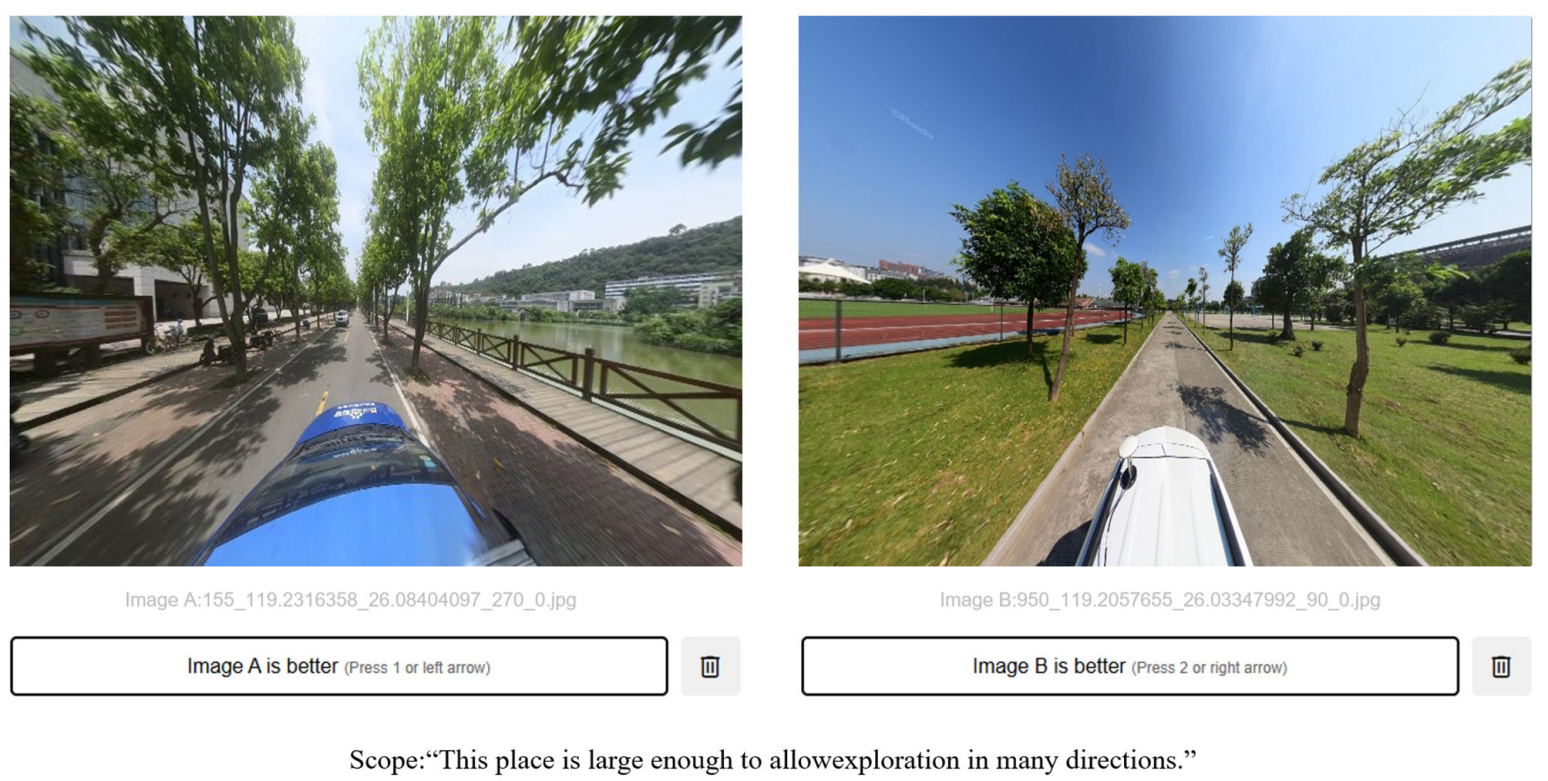
Trueskill algorithm interface.

In order to construct a training dataset with relatively stable and uniform standards, the Trueskill algorithm is used for labeling the training samples. Trueskill was originally applied to Microsoft's game ranking system, which is a Bayesian scoring system that generates overall ranking scores based on the cumulative results of two-on-two matches and iteratively updates the scores based on the most recent each two-on-two matches to generate new winners' and losers' rankings, thereby converting the binary comparison results into continuous ranking scores (Minka et al., 2018). In this study, the logic of Trueskill's algorithm was used to design web pages available for comparison operations, and the back-end of the web pages were made available for participants to compare their strengths and weaknesses on the front-end of the web pages by randomly selecting street images from 2 sample points in the training dataset. In this study, the comparison modules were established according to the PRS-11 scale for each of the four categories of distance, coherence, scope, and attractiveness, and based on the winners and losers of each round of comparison, the ranking results of all the training sample points in each of the four modules were iteratively recorded and the corresponding ranking scores were output. For the study, a random selection of 2,980 campus SVIs (~20%) was made (Figure 3). In October 2024, a total of 114 student evaluations were gathered through a 2-week online survey, consisting of 54 females and 60 males, with ages ranging from 20 to 24 years (mean age: 21.7 years). Participants were asked, based on the PRS-11 questionnaire, to choose the image that most closely matched the one described on our platform (Figure 3). Finally, we experimented to predict the recovery quality scores using the CNN-BiLSTM model and the overall campus SVI recovery quality scores based on questionnaire data from 2,980 randomly selected campus SVI, of which 20% were used as the test set and 80% as the training set. Meanwhile, we assessed the validity of the model using mean square error (MAE) and coefficient of determination (*R*^2^), with *R*^2^ ranging from 0.841 to 0.964. MAE ranging from 0.040 to 0.109.

#### 2.4.7 Matlab visual entropy and color complexity calculation

Matlab software, known for its strong compatibility, was selected to compute Visual Entropy and Color Complexity from the image data. In landscape visual perception research, Visual Entropy is commonly employed to quantify the overall complexity of an image, while Color Complexity is used to evaluate the impact of color in landscape configurations on human perception levels. Equation 1 (Stamps III, 2004) and Equation 2 (Zhou et al., 2015) demonstrate the calculation principles of Visual Entropy and Color complexity, respectively.


(1) $$\begin{array}{l} H\left(x\right)=-\overset{n}{\underset{i=1}{\sum }}P\left(a_{i}\right)*\log P\left(a_{i}\right) \end{array}$$

(1) where: *n* is the number of regions or units with significant boundaries, *i* is the divided region, *P*(*a*_*i*_) is the probability that region *a*_*i*_ (*i* = 1, 2, …, *n*) the probability of occurrence, and the amount of information given as *H*(*x*) denotes the total amount of information generated for the entire visual object consisting of *n* regions.


(2) $$\begin{array}{l} \begin{array}{c} C_{K}=-\overset{m}{\underset{i=1}{\sum }}n_{i}\log\left(\frac{n_{i}}{N}\right) \end{array} \end{array}$$

*C*_*k*_ denotes the complexity of the spatial distribution features of a certain color; *m* denotes the number of different connected regions in the general set; *n*_*i*_ denotes the number of pixels in the *i*-th connected region; and *N* represents the total number of pixels of that color.

#### 2.4.8 Extreme Gradient Boosting Tree (XGBoost) model and SHapley Additive exPlanation (SHAP) interpretability analysis

This study uses the XGBoost algorithm to create a regression model, applies Bayesian optimization, and then analyzes the results with the SHAP method to explore how the audiovisual perception indicators of the campus landscape relate to the quality of attention restoration. Because of its superior parallel processing capabilities and capacity to handle complicated non-linear interactions, XGBoost can successfully tackle machine learning problems. Possible issues with regression modeling overfitting (Chen and Guestrin, 2016). In addition, nowadays, various researches utilize various machine learning methods such as random forest (Feng et al., 2024), regression tree (Kesgin Atak, 2020), and XGBoost (Chen and Guestrin, 2016), among which XGBoost has the advantages of high efficiency, performance, and superior regression results that are widely utilized by researches (Ma et al., 2024). However, Machine Learning and Artificial Intelligence (ML/AI) was previously considered as a black-box approach, and recent advances in scalable AI (XAI) have led to increasing interpretability. In particular, the local interpretation approach of SHAP (SHapley Additive exPlanations) provides research with the flexibility to model, explain and visualize complex phenomena and processes (Chen J. et al., 2024). The underlying principle is illustrated in Equation 3:


(3) $$\begin{array}{l} g(z^{\prime })=\varphi_{0}+{\sum_{i=1}^{M}\varphi }_{i}{z^{\prime }}_{i} \end{array}$$

*g*(*z*')denotes the predicted value of *z*' the influence of the sentiment index of landscape elements, φ_0_denotes the average indicator of the composition of landscape elements, *M* denotes the number of variables in the model, and denotes the SHAP value of the *i*-th study indicator. For each prediction, the Shapley value is calculated by computing the average marginal contribution of all possible combinations of explanatory variables. Finally, SHAP performs a 50% discount cross-validation.

## 3 Results and analysis

### 3.1 Distribution of campus audiovisual perception indicators and attention restorative quality indices

**Figure 4:**
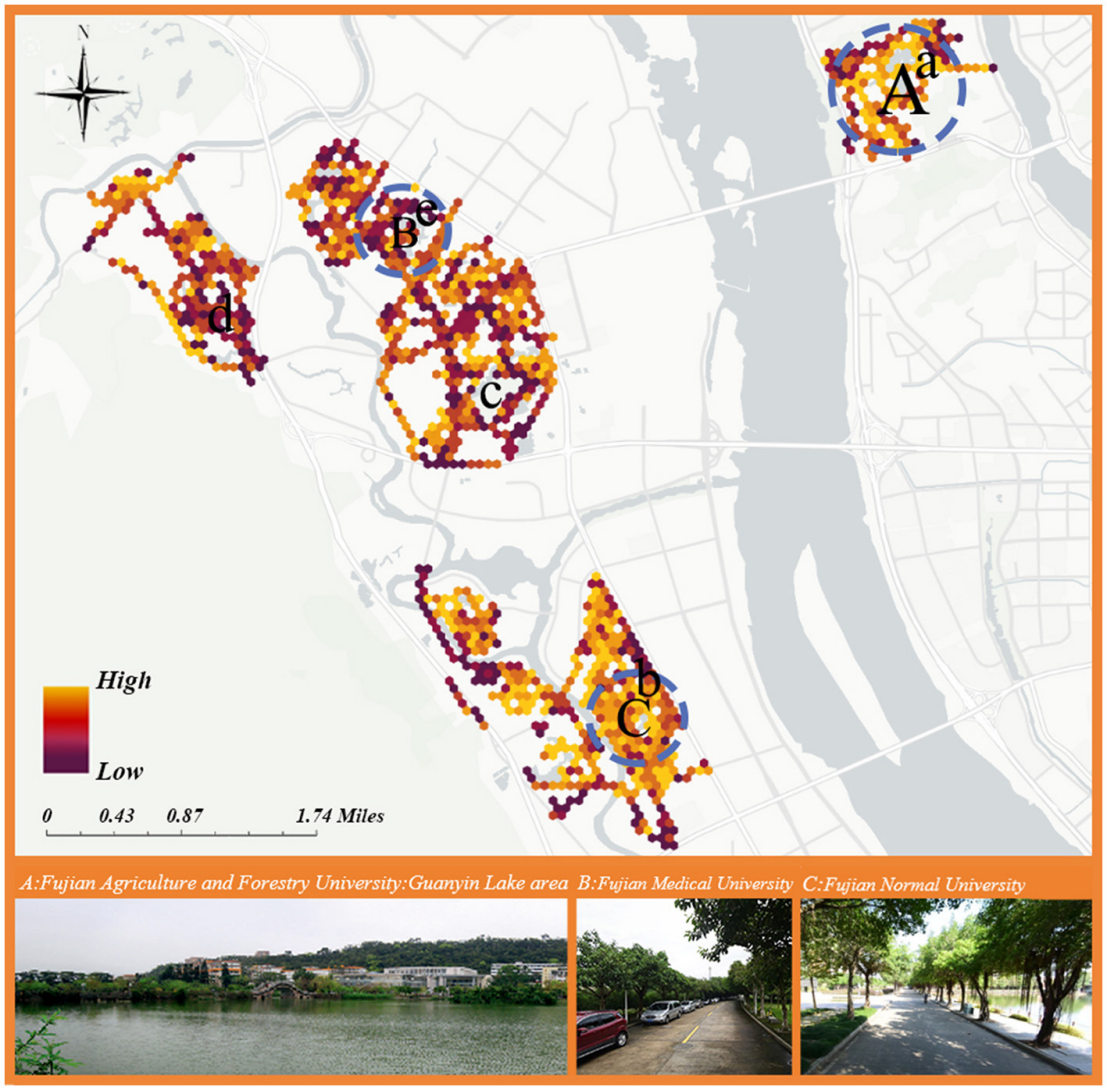
Distribution of quality index of attention recovery in college students.

Figure 4 maps campus restorative quality predictions from the CNN-BiLSTM model (96.43% accuracy) using Trueskill-generated PRS-11 scores averaged across 80-meter hexagons. The hexagonal grid enables continuous spatial visualization while minimizing localized interpretation errors, with lighter areas indicating higher restoration quality. This approach supports targeted zone renewal through regional condition analysis.

Figure 4 shows higher restorative quality in campuses with rich humanistic heritage (e.g., Fujian Agriculture and Forestry University, Fujian Normal University), particularly around Guanyun Lake. Waterfront spaces enhance restorative effects through cooling microclimates and meditation opportunities (Burmil et al., 1999), contrasting with unexpectedly low-quality water landscapes at Fuzhou University and Fujian Medical University. Campus sports fields (e.g., FAFU-a, FNU-b) demonstrate superior attention recovery through stress-relieving outdoor activities (McCormack et al., 2010), while playgrounds at Fuzhou University-c, Minjiang University-d, and Fujian Medical University-e show weaker restorative performance.

**Figure 5:**
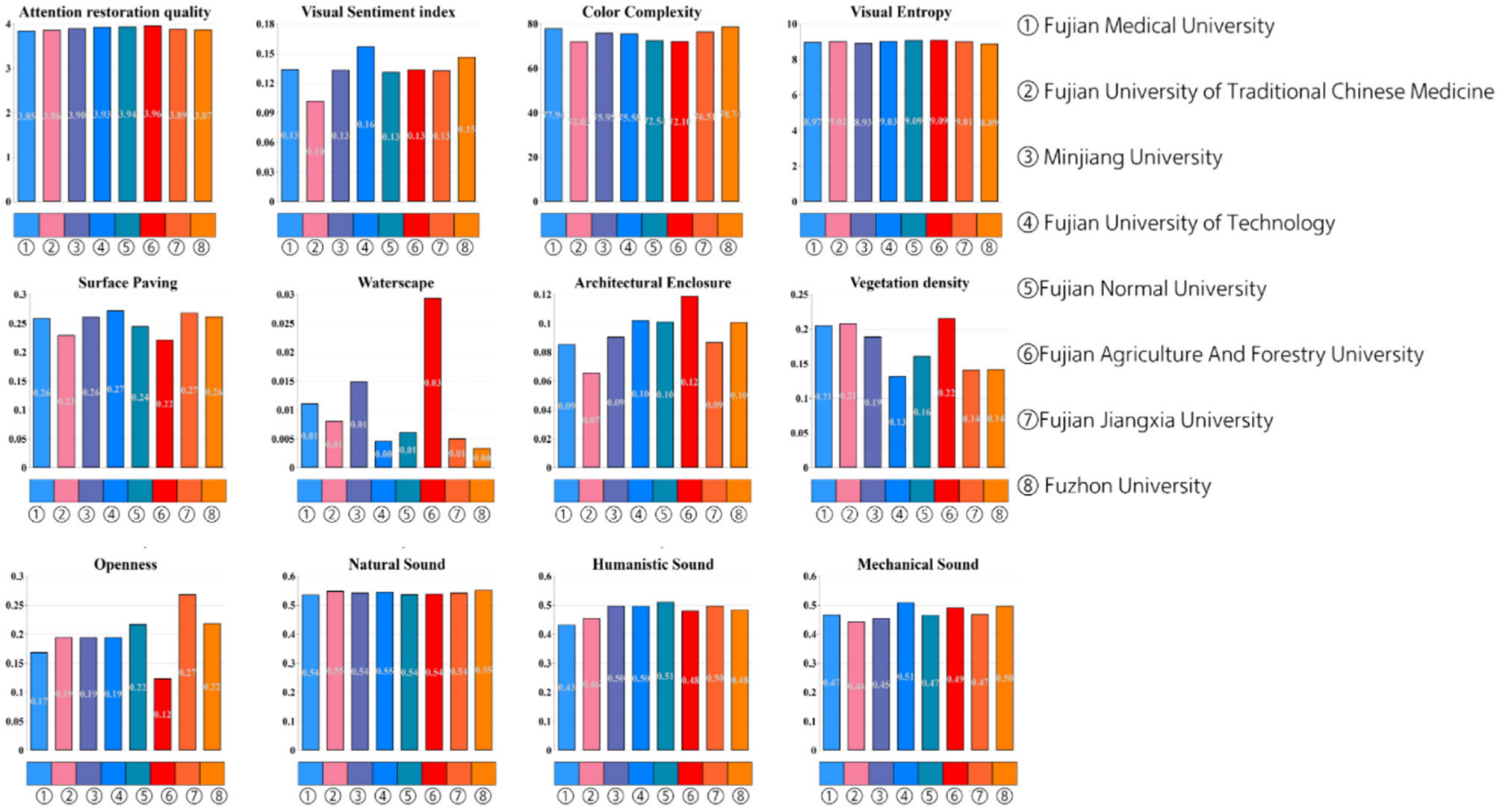
Average values for attention restoration and audiovisual elements across various institutions in Fuzhou.

Based on the data in Figure 5, the attention restoration quality of universities in Fuzhou exhibits distinct variations. Fujian Agriculture and Forestry University (3.961) and Fujian Normal University (3.937) demonstrate the highest restorative efficacy, correlating with their elevated vegetation density (0.216 and 0.161, respectively) and higher proportions of natural sound (0.538 and 0.537), suggesting the positive impact of ecological elements on cognitive recovery. In contrast, Fujian Jiangxia University (3.886), despite having the greatest openness (0.268), shows potential inhibitory effects from its high traffic sound proportion (0.638). Notably, Fujian University of Technology (3.929) achieves optimization through stronger architectural enclosure (0.272) and musical sound elements (0567), reflecting an alternative humanistic acoustic strategy. Visual complexity metrics reveal nuanced relationships: Minjiang University's moderate visual entropy (8.933) and Fuzhou University's high color complexity (78.735) indicate potential synergies between controlled visual stimuli and spatial design. The inverse relationship between mechanical sound (0.468) and traffic noise (0.548) at Fujian Medical University, compared to Fujian University of Traditional Chinese Medicine (0.443 mechanical sound vs. 0.523 traffic sound), highlights the critical balance of acoustic components in medical institution environments. These patterns collectively emphasize the multidimensional interaction between biophilic design, auditory composition, and spatial configuration in shaping restorative campus landscapes.

### 3.2 SHAP characteristic importance analysis

**Figure 6:**
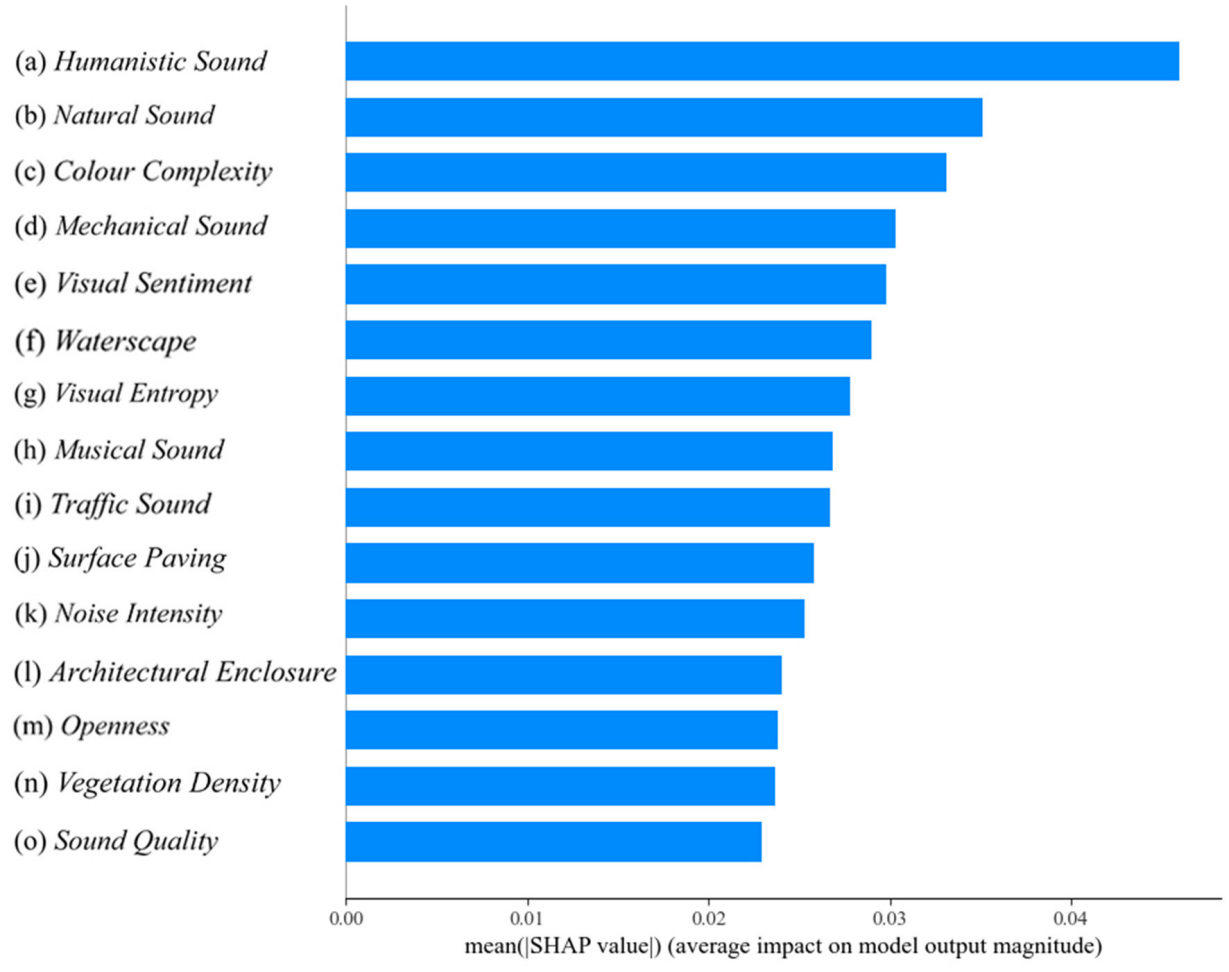
The importance of features in SHAP(*p* < 0.001).

**Figure 7:**
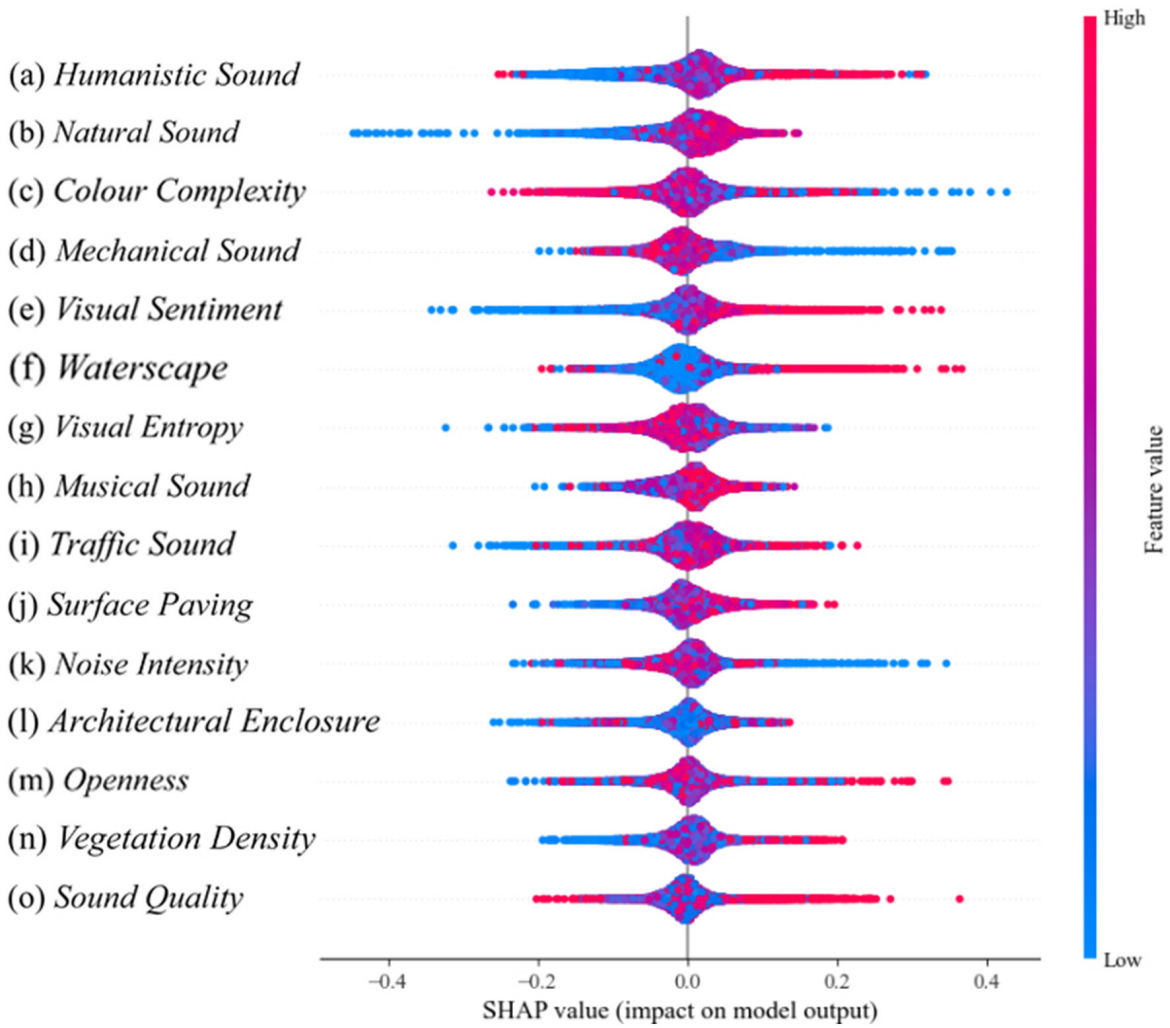
Characteristic swarm diagram in SHAP (*p* < 0.001).

In this study, XGBoost modeling and SHAP analysis were used to explore the effects of campus landscape environmental factors and sound indices on the quality of college students' attentional recovery. Various machine learning methods were applied in the study, such as random forest (Feng et al., 2024), regression tree (Kesgin Atak, 2020), and XGBoost (Chen and Guestrin, 2016). People widely use XGBoost due to its efficiency, powerful performance, and excellent regression results (Chen J. et al., 2024). As shown in Figures 6, 7, the visual markers highlight the importance of the various research metrics on the quality of attention recovery and provide an understandable overview of the research results. In Figure 6, the human voice contributes the most to the model.

The importance of humanistic sound in this study is high, but some of the humanistic sound have a negative impact, which may be related to their ability to moderately stimulate the senses, enhance the social atmosphere, and optimize the experience of the environment. The effects of color complexity show both positive and negative aspects, which are related to human processing mechanisms of visual information. The visual sentiment, the higher the proportion of waterscape, the greater the positive impact on the model is illustrated in Figure 7. The indicators of color complexity, visual entropy, music sound, traffic sound, and architectural enclosure show a linear relationship, which may be related to the scene in which they are located. Therefore further analysis is needed. In addition, for example, indicators such as surface paving and vegetation density when the eigenvalue is high, a few points show a negative impact. This may be related to the rationality of its design, the overall harmonization of the environment, and the match between personal preferences and the use scenario.

In conclusion, a fundamental analysis was carried out on the various research indicators, as detailed in Sections 3.1 (Distribution of Campus Audiovisual Perception Indicators and Attention Restorative Quality Indices) and Section 3.2 (SHAP Characteristic Importance Analysis), focusing on both their spatial distribution and the significance of their characteristics. Although certain metrics revealed distinct patterns of feature importance, the majority of the study's metrics—such as color complexity, visual entropy, and musical sound—demonstrated intricate interactions. Nonetheless, these metrics significantly contribute to the model, though the nature of their relationship with sentiment indices of varying values requires further clarification. To address this, partial dependency graphs will be employed in the experiment to delve deeper into their interconnections.

### 3.3 SHAP partial dependency graph analysis

**Figure 8:**
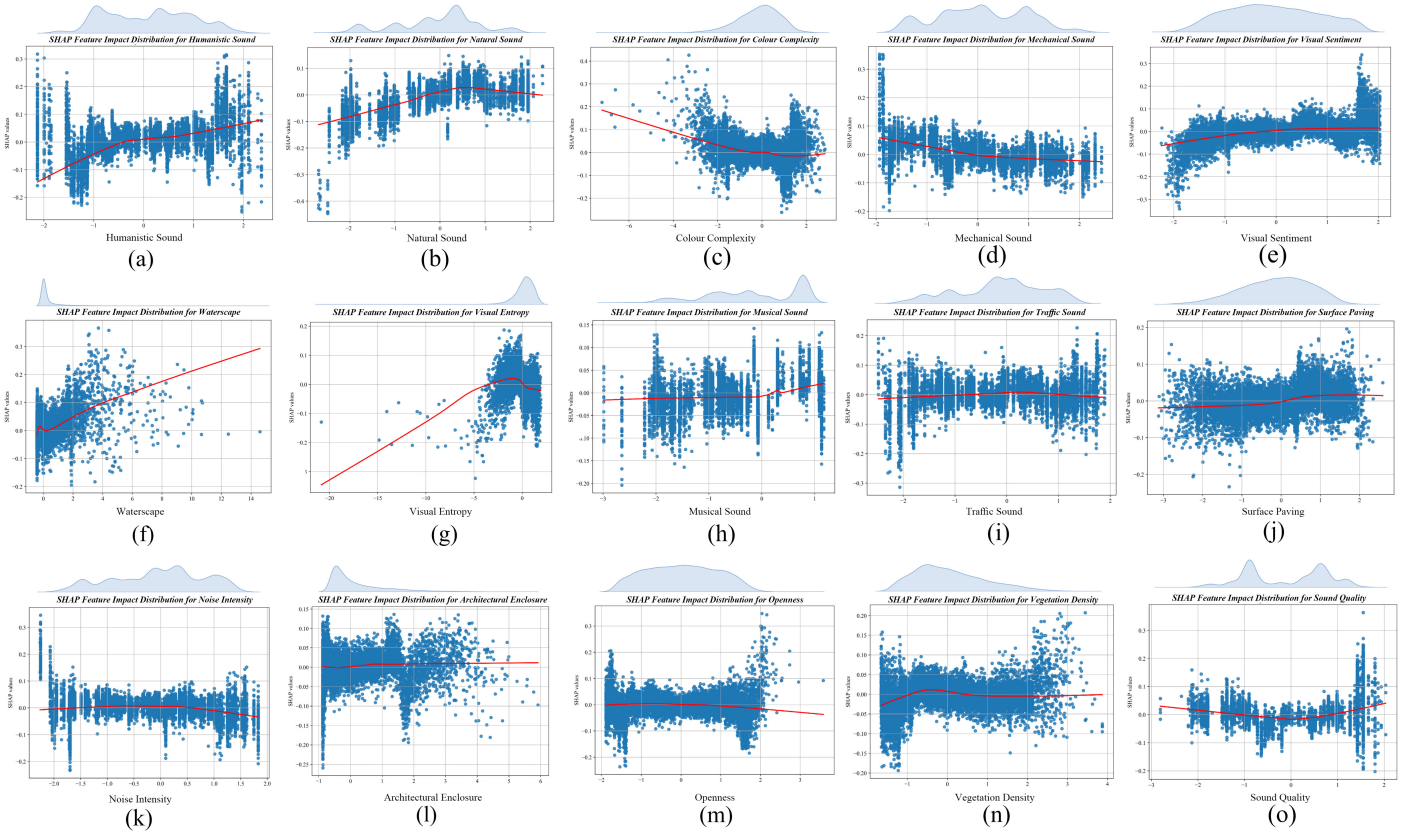
SHAP feature edge density and dependency diagram. **(a)** Humanistic sound. **(b)** Natural sound. **(c)** Color complexity. **(d)** Mechanical sound. **(e)** Visual sentiment. **(f)** Waterscape. **(g)** Visual entropy. **(h)** Musical sound. **(i)** Traffic sound. **(j)** Surface paving. **(k)** Noise intensity. **(l)** Architectural enclosure. **(m)** Openness. **(n)** Vegetation density. **(o)** Sound quality.

In Figure 8, the variables are ranked based on their contribution values presented in Figure 7, with the inclusion of fitted curves and marginal density plots for the eigenvalues. This approach facilitates a more intuitive exploration of the distributions of the eigenvalues. Figures 8a–c mainly represent the most important influential variables. Figure 8a illustrates that some of the eigenvalues of humanistic sound between −1 and −0.5 show negative contributions, however, half of the eigenvalues greater than −0.5 show positive correlations, which may be due to a combination of sound type, visual characteristics, individual preferences, and model complexity. Figure 8b presents natural sound, which show positive correlations between −0.25 and 2, peaking at 0.5, and negative correlations above 2. It's possible that the experience deteriorates due to excessive sound intensity, which exceeds the human comfort threshold for natural sounds. Figure 8c depicts the color complexity, whose value distribution is bounded by 0, and < 0 presents a positive contribution. Its positive contribution portion is mainly clustered between −4 and 0, suggesting that keeping complexity between −4 and 0 enhances the contribution to the positive aspects of the Attention Recovery Quality Index.

Second, Figures 8d–g shows the important variables. Figure 8d is the mechanical sound, which has a uniform distribution density from the edges of its eigenvalues and lacks significant peaks or dense areas, but presents a positive correlation with values <0, which indirectly confirms a possible non-linear relationship or threshold effect between the variables. For a more precise interpretation, this can be further analyzed in conjunction with the interaction plots in the next chapter. According to Figure 8e visual sentiment presents a positive correlation at >0, but its feature edge density is symmetrically distributed with 0 as the boundary. It shows that the feature values of positive and non-positive perceptions are statistically balanced, with neither obvious bias nor clustering effect in specific areas. Figure 8f waterscape, on the other hand, is rising to present a positive effect, but the effect becomes unstable and presents a complex dynamic trend after exceeding the threshold value of 5. Figure 8g visual entropy eigenvalue density is mainly clustered between −5 and 2, showing rising positive contribution, and negative contribution after >0, indicating that the increase of visual entropy can help attention recovery within a moderate range.

Finally, Figures 8h–o shows the less significant variables. Figure 8h shows that the sound of music presents a positive contribution beyond 0.25 with a value marginal density mainly distributed around 1, which implies a threshold for school music. Figure 8i shows that traffic sound presents a little positive contribution mainly between −1 and 1. Figure 8j shows that the surface paving presents a positive correlation at >0. Figure 8k shows that the SHAP value of noise intensity mainly hovers around 0 and exhibits a positive correlation within the eigenvalue range of −1.5 to 0.5, suggesting that moderate noise intensity may positively affect the target variable, but the overall effect is weak and confined to a specific range. Figure 8l shows that the fitted curves of architectural enclosure always show positive correlation, but the contribution value hovers around 0, indicating that its positive effect on the target variable is relatively limited. Meanwhile, the characteristic edge densities are mainly concentrated between −1 and 1, reflecting a more concentrated distribution of architectural enclosure, which does not show a significant effect of extreme values on the SHAP values. Figure 8m shows that openness shows a negative correlation after >1, and its value edge density is mainly concentrated in positive contribution. Figure 8n shows that the values of vegetation density are mainly clustered between −1 and 1 and present a stable positive contribution to the target variable in this range. However, when the value exceeds 3, it exhibits instability despite continuing to present a positive contribution, suggesting that too high a vegetation density may trigger complex dynamic effects that become unpredictable or have increased volatility on SHAP values. Figure 8o shows that sound quality presents a negative effect between −1 and 1.

In summary, most indicators exhibit complex non-linear effects and coupled correlations between their geographic spaces. Therefore, in the next section, we use SHAP interaction value analysis to further reveal how the interactions between different indicators affect the quality of attention recovery among college students.

### 3.4 Analysis of SHAP interaction values

**Figure 9:**
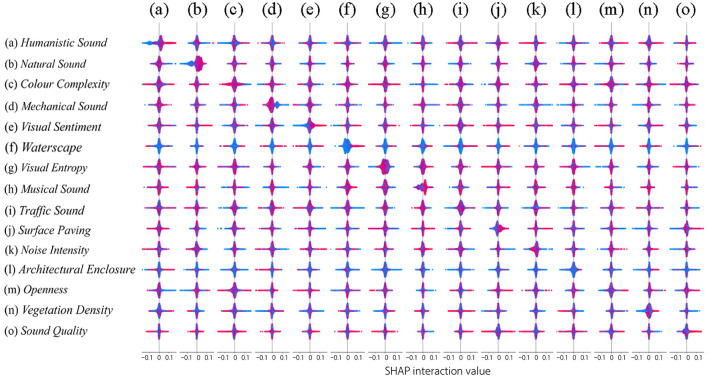
SHAP interactive analysis diagram.

As illustrated in Figure 9, a SHAP interaction plot is presented to explore the mutual interactions among variables, thereby aiding in the analysis of their coupling mechanism within the model. The findings reveal that:

(1) Most of the indicators of the campus show a more balanced effect of influence when interacting (the characteristic points are evenly distributed around the zero line), but individual indicators interact with different effects on the quality of attention recovery. For example, color complexity was the most negatively contributing of the campus environment metrics, but color complexity positively affected the quality of attentional recovery when interacted with sounds of people moving, natural sounds, pavement rate, water view, and openness (more feature points to the right of the zero line).
(2) Although the overall contribution of the music sound and surface paving metrics to the model was negligible or even slightly negative, Figure 9 shows that when interacting with metrics such as greenness and surface paving, openness led to predominantly positive interaction values. When interacting with metrics such as waterscapes and surface paving, the music sound metrics mainly lead to positive interaction values. This suggests that music sound metrics can significantly and positively influence the quality of attentional restoration in specific situations (Shen, 2023). Where appropriate surface paving has a positive contribution when interacting with traffic sound, noise intensity and mechanical sound, but surface paving can have a positive contribution when interacting with natural sound and musical sound. Therefore, different paving materials as well as the degree of paving may have an impact on people's appreciation of the soundscape (Ma et al., 2024).
(3) Finally, the campus in this study area is dominated by a waterscape as the core of the campus, and the waterscape and vegetation density showed the most significant threshold ranges in the interaction plot. It further suggests that waterscape and vegetation density elements may be the key to campus landscape configuration. Their optimal values may significantly influence changes in the quality of attention restoration. For example, waterscape and vegetation density interacted with humanistic and musical sound to produce positive contributions, and these sounds and vegetation could enhance the soundscape eventfulness of the lake body zone. Where vegetation density also showed positive effects when interacting with architectural enclosure.

This figure shows interaction effects that can lead to a better understanding of issues in campus audiovisual environments and college students' attentional preferences by identifying metrics with significant positive or negative tendencies in interaction scenarios. In addition, it should be noted that similar metrics may contribute differently between campuses due to different campus landscape plans.

## 4 Discussion

### 4.1 Novel perspectives on key indicators influencing the quality of attention restoration among college students on campus

#### 4.1.1 The quality of green space restoration at liberal arts and agricultural universities is better than at science and engineering universities

This study reveals the significant impact of diverse audiovisual elements on attention restoration among college students, with cultural sounds, environmental sounds, and visual complexity emerging as key factors. Research indicates that preferences for natural environments vary significantly across regions and groups (Gasco et al., 2020). In artificial environments, vegetation density, building enclosure, and paved surfaces contribute little to attention recovery. These environmental factors, which are primarily influenced by human activities, are often perceived by individuals as lacking uniqueness and failing to meet their expected needs (Zhang et al., 2023). Additionally, excessive vegetation density may trigger attention dispersion, suggesting that unplanned wilderness landscapes may exacerbate the instability of attention recovery effects. Research suggests that optimizing artificial environment design by emphasizing uniqueness and innovation can promote creativity and attention recovery. Such designs can leverage the “soft charm” mechanism in Attention Restoration Theory (ART) by employing artificial soundscapes (e.g., simulated tea-picking sounds). Such soundscapes can trigger cognitive labeling of “environmental background sounds” based on their ecological effectiveness—the compatibility between sound sources and cultural semantics—thereby effectively reducing individuals' active monitoring needs. This effect synergizes with the “compatibility” dimension of Attention Restoration Theory (Kaplan, 1995; Kexin et al., 2024).

The geographical distribution of students' attention recovery quality indicates that universities with a focus on humanities and agricultural research, such as Fujian Normal University and Fujian Agricultural University, exhibit superior attention recovery quality. Conversely, universities with a concentration on science and engineering, such as Fuzhou University, Fujian University of Science and Technology, and Fujian Medical University, exhibit inadequate attention recovery quality, which is associated with the disparate dissemination of cultural genes. Fuzhou Agricultural and Forestry University (FUAF) exemplifies its “farming culture” through its distinctive landscapes, including the China Famous and Excellent Plant Garden and the Agricultural Culture Corridor. These features offer aesthetically pleasing ecological interfaces and facilitate “five-sense immersion” through participatory farming experiences, such as tea picking and mushroom cultivation (Kexin et al., 2024). This culturally rich landscape design closely aligns with the “compatibility” principle in Attention Restoration Theory (ART). The influence of human and natural sounds, in conjunction with color complexity, on landscape perception is a subject of considerable interest. This influence is primarily attributable to the substantial impact of natural events and human activities on environmental perception (Diepstraten and Willie, 2021). According to the principles of color psychology, the color of a given environment has the capacity to influence the emotional state of the individual (Zailskaite-Jakšte et al., 2017). Specifically, the sounds produced by human activity within informal learning environments (ILE) have the potential to induce a state of relaxation, to create a phenomenon of sound masking, and to enhance the motivation of the individual to engage in the learning process (Zhang et al., 2024). Agricultural and forestry schools manifest boundary effects, which are evident in transition zones between natural and human-made landscapes. These zones include, but are not limited to, the areas between fields and laboratories, as well as the various agricultural zones (e.g., farmland, forestland, etc.). The existence of these boundaries fosters the cultivation of unique “spatial” and “ecological” perceptions, thereby establishing a psychological barrier between academic and practical activities. This psychological barrier enables students to refocus their attention and restore emotional balance in complex work environments.

Conversely, universities with a strong emphasis on science and engineering, such as Fuzhou University, Fujian University of Science and Technology, and Fujian Medical University, demonstrate substandard attention recovery quality. This phenomenon may be associated with internal planning, cultural landscape design, and vegetation layout. In contrast, the study revealed that augmenting plant density did not yield a substantial enhancement in recovery quality, thereby contradicting the findings of prior research (Bornioli et al., 2018). The SHAP analysis results (Figures 6–8) indicate that mechanical noise has a significant negative contribution to attention recovery, with its impact intensity exceeding that of traditional environmental factors such as vegetation density. This effect may be related to the persistent mechanical noise commonly found in densely populated laboratory areas of science and engineering universities. Notably, the interaction between mechanical noise and color complexity exhibits a non-linear characteristic (Figure 9), which explains why simply increasing plant density is ineffective in improving recovery quality at science and engineering universities. This finding forms a structural contradiction with the “escape” dimension in attention recovery theory: mechanical noise, as a derivative signal of laboratory activities, continuously triggers individuals' cognitive associations with academic stress (Zhang et al., 2023). The strong coupling between soundscape elements and spatial functions leads to a “cognitive-environmental” negative feedback loop in science and engineering universities—laboratories are both the core areas of knowledge production and the primary sources of attention depletion. This necessitates that campus restoration design must transcend traditional landscape planning paradigms by employing soundscape masking (e.g., introducing wide-frequency environmental sound barriers) and spatial buffer zones to disrupt the spatiotemporal continuity of mechanical noise.

#### 4.1.2 Environmental optimization path under the SHAP interaction mechanism

(1) Synergistic optimization of audiovisual elements: In the context of campus environment design, it is imperative to consider the interplay between color and soundscapes. In areas with high pedestrian traffic, such as plazas and walkways, the dynamic integration of color complexity—for example, multi-tonal ground paving and varying vegetation layers—with natural soundscapes, including flowing water and bird songs, or moderate human activity sounds, has been shown to balance the potential distractions caused by monotonous colors on attention recovery. This, in turn, creates an immersive sensory experience. Furthermore, in areas characterized by abundant vegetation, such as lakesides or tree-lined pathways, the implementation of low-intensity background music, including natural sound effects or gentle melodies, can be considered. The positive interaction between music and vegetation density has been demonstrated to enhance the richness and eventfulness of the soundscape while effectively mitigating the negative impacts of mechanical noise. This results in a sound environment that harmoniously blends relaxation and vitality.
(2) Adaptive design of ground paving materials: Select paving materials based on functional zoning requirements: In areas with significant traffic noise (such as school gates and vehicle lanes), use sound-absorbing and permeable paving materials (such as porous asphalt and ecological bricks) to suppress noise propagation through the interaction between material properties and traffic noise. In areas dominated by natural soundscapes (such as waterfront walkways and meditation gardens), use wooden or gravel paving to leverage their synergistic effects with natural and musical sounds, creating an immersive “walkable soundscape experience.” Additionally, through visual guidance design of paving textures (e.g., circular radiating geometric patterns or color gradients), dynamic visual responses are created with open space vistas, enhancing the intuitiveness of directional perception while mitigating the potential distractions caused by spatial enclosure on attention recovery.
(3) Threshold-based configuration of water bodies and vegetation density: Using core water bodies (such as artificial lakes) as acoustic landscape anchor points, extend stepped vegetation buffer zones outward (transitioning from high-density trees to low shrubs). Through the interactive threshold effects of water body and vegetation density, balance acoustic landscape screening and visual permeability. Simultaneously, in enclosed building areas (such as courtyards, corridors), integrate vertical greening with dynamic water features (such as cascading walls and mist sprays). By leveraging the synergistic effects of building facades and vegetation, create composite spaces that combine microclimate regulation with soundscape therapy functions, thereby enhancing the immersive quality and comfort of the environmental experience.

### 4.2 Planning and design recommendations for campus audiovisual environments

#### 4.2.1 Soundscape optimization and integration of humanities activities

In the study of attention recovery campus environments, it would be more effective to create an attention recovery space that is consistent with spatial preferences. This process must prioritize the cultivation of a unique campus character in the landscape, while enhancing the attention recovery and emotional perception of college students without destroying the inherent cultural appeal of the campus. Figure 8 shows that humanistic and natural sounds have a strong, albeit slightly fluctuating, effect on the quality of attention recovery. The next positive contribution is presented when plant density interacts with building enclosure. This observation is slightly different from the previous ones, where there were studies that showed a significant positive effect of green space on recovery (Tang and Long, 2019), where there were also studies that showed a significant negative effect of green space on recovery (Chen Z. et al., 2024). The influence of this situation is shaped not only by plant density and building enclosure but also by several other factors, including design quality, spatial usage, individual needs, and environmental noise. In the realm of landscape architecture, it is crucial to explore how green spaces contribute to enhancing attentional restoration, with a particular focus on their interplay with auditory elements. The findings of this study highlight the substantial role that optimal soundscapes and enriching visual experiences play in enhancing sentiment recovery and alleviating stress (Xu et al., 2024). Moreover, the combined interaction of auditory and visual stimuli appears to offer more substantial recovery benefits than either sensory input on its own. Carefully crafted audiovisual settings, especially those featuring balanced natural landscapes and calming soundscapes, can significantly mitigate academic pressure, ease cognitive fatigue, and enhance emotional health (Hong et al., 2019; Guo et al., 2022; Liu et al., 2022; Pande et al., 2024). Previous studies have highlighted environmental disparities across various campuses, leading to the proposal of strategies designed to improve perceptual recovery and emotional well-being of college students through the adjustment of audiovisual element thresholds. This research, in particular, identifies that attentional recovery perceptions were notably lower in polytechnic institutions. This may be attributed to factors such as the noise from laboratory environments and the relative absence of humanistic elements in the campus design (Chen J. et al., 2024). This can be done by the fact that in learning spaces such as laboratories, classrooms and study rooms, the interference of external noise can be reduced by optimizing the building design and adding soundproofing measures, such as the use of sound-absorbing materials, soundproof windows, and so on.

As shown in Figure 8, having water landscapes greatly affects the sounds around us, with natural sounds and plants making the area more lively. Consequently, the organization of activities such as art exhibitions, concerts, and festivals in the vicinity of the water body has been identified as a strategy to promote students' physical and mental relaxation, alleviate academic pressure, and enhance the humanistic charm of the campus and facilitate social interaction. In polytechnic institutions, the absence of vegetation design, as well as activity events surrounding the water features, results in a suboptimal restoration effect on the water features' attention. The restoration areas with minimal attention in liberal arts institutions and agricultural and forestry institutions are predominantly located in the campus periphery. To enhance this phenomenon, it is recommended that a variety of landscape elements, including small gardens, ponds, sculptures and walkways, be incorporated into these areas. Research demonstrates that this approach enhances the visual experience and boosts the environment's restorative function. Furthermore, the acoustic environment is also a pivotal factor in enhancing the recovery of attention, thus music nodes can be designed in the peripheral areas to utilize the soundscape to enhance the sense of relaxation and recovery in the space.

#### 4.2.2 Adaptive management of intelligent perceptual systems

Pouso suggest that the health advantages of blue-green spaces become evident only when individuals engage with them directly (Pouso et al., 2021). Consequently, before engaging in landscape planning, it is essential to establish a comprehensive research framework that evaluates and classifies landscape resources across various campuses. This framework should provide data support for addressing specific challenges, such as areas with high pedestrian traffic density. To make timely adjustments, it is crucial to monitor factors such as pedestrian flow, environmental shifts, and user feedback (Yang et al., 2023). Most of the current machine learning models, on the other hand, are for the analysis of a fixed-point image, for this reason, this study considers applying the cross-modal distillation paradigm to construct a sentiment perception model (CSV-T4SA), which can be adapted to new data distributions and features through continuous learning and updating. This allows the model to maintain its sophistication and accuracy to serve the field of social media sentiment analysis in the long run. The model can be implemented on campus forums, bulletin boards, and other relevant social platforms to quantitatively analyze various forms of information, thereby facilitating the development of tailored, real-time management strategies. Furthermore, the results of the spatial analysis can be combined with public input and management strategies aimed at promoting active engagement from both students and faculty (Zhu et al., 2023). Special attention should be given to the emotional interactions between college students and the surrounding landscape in regions demonstrating lower levels of attention recovery. By adopting this approach, responsiveness to the needs of university students is enhanced, while the overall management and upkeep of attention restoration quality in campus landscapes is improved. This not only contributes to the well-being of students but also supports the long-term sustainability of urban ecosystems.

### 4.3 Application and contribution of the framework for assessing audiovisual environment and attentional restoration on campus

This study primarily uses social media data and the PRS-11 questionnaire for image quantification. The specific process involves mapping the average values of the four recovery perception scores from the PRS-11 questionnaire onto a hexagonal grid with an 80-m radius. The study employs techniques such as XGBoost and SHAP importance analysis to delve deeper into data insights. Current research has primarily focused on green spaces and urban areas (Wei et al., 2022), while studies on the effects of soundscapes on attention recovery in campus environments remain limited. Recently, innovative data sources for soundscape assessment have been increasingly introduced, such as social media, complaint reports, and three-dimensional urban models (Aiello et al., 2016; Stoter et al., 2020; Tong and Kang, 2021). Research indicates that SVI data holds significant advantages in campus audiovisual environment assessment, enabling large-scale, low-cost evaluations. This study employs the SHAP framework for quantitative analysis, focusing on feature importance rankings and dependency relationships to reveal the model's decision-making mechanisms. Additionally, the newly introduced feature value marginal density distribution aids in comprehensively understanding how variables influence the model. The interaction value analysis in Figure 8 illustrates the relationships between variables in the audiovisual environment, providing guidance for campus design. The study emphasizes the advantages of machine learning in analyzing complex datasets and proposes a structured approach. By mapping repair perception scores onto a hexagonal grid, spatial performance can be easily observed, with lighter-colored areas indicating higher repair quality, ensuring smooth results.

### 4.4 Limitations of the research and future directions for exploration

This study integrates machine learning methodologies with social media datasets to investigate the impact of campus audiovisual environments on attention recovery. Although social media has gradually become a powerful tool for academic research (Hodorog et al., 2022), data security and regulatory restrictions may hinder comprehensive research on the perceived experiences of teachers and students of different age groups in campus environments (Cortis and Davis, 2021). Therefore, to include a broader student population, future research should incorporate field survey data to enhance the external validity of the study. The present study primarily collected SVI data during the summer months, yet it did not engage in explicit temporal analysis of social media or text data. It is important to note that summer offers optimal vegetation visibility; however, the audiovisual environment on campuses (e.g., vegetation density, water body usage, and human activity patterns) may exhibit significant differences during non-summer seasons (e.g., autumn, winter, and spring). Such variations may impede the generalizability of the study's findings across seasons. For instance, increased leaf fall in autumn, vegetation dormancy in winter, or vegetation growth in spring may have the potential to influence the effects of attention recovery. Consequently, the summer data set in this study may not have fully captured such dynamic changes. Furthermore, the summer period encompasses both active academic terms (June and September to October) and vacation periods (July to August). During these periods, there may be notable variations in campus activity levels, pedestrian density, and soundscape characteristics. However, this study did not differentiate between specific time points for image collection during the summer, which limits the representativeness of the “typical” academic experience. The audiovisual dimensions of the campus environment are significantly influenced by seasonal characteristics. For instance, dense vegetation during the summer months may significantly enhance an organism's recovery potential, as postulated by attention recovery theory and biophilia theory. Conversely, sparse vegetation during winter may reduce this effect. Furthermore, the negative impact of traffic noise on recovery (social recovery theory) may be particularly pronounced during peak hours (Ratcliffe, 2021). Furthermore, the circadian rhythms of the campus environment, such as the daytime bustle and nighttime tranquility, may significantly alter perceived experiences and the quality of attention recovery. It is imperative to acknowledge that campus landscapes and infrastructure have undergone continuous changes from 2021 to 2023, and these dynamic changes may not be fully reflected in the application phase of the study (2024 and beyond). Moreover, the data from 2021 to 2022 were influenced by the ongoing pandemic and modifications to prevention and control policies. These adjustments may have led to alterations in summer campus usage patterns, such as a reduction in activities or the implementation of specific preventive measures. Consequently, the representativeness of the data may have been compromised. In summary, while the static analysis method employed in this study is well-suited for large-scale research, its methodological nature precludes the capture of the aforementioned dynamic effects. This may result in biases in the estimation of the relationship between soundscape characteristics and attention recovery effects. Future research could collect longitudinal data across seasons and different time periods to clarify the impact of time dependency on perceived recovery. The findings of this study may be influenced by the unique subtropical climate of Fuzhou, which significantly affects vegetation types and seasonal change patterns (Diepstraten and Willie, 2021). To enhance the generalizability of the results, replication studies in different climate zones are particularly necessary.

In the context of soundscape prediction models, the present study first focuses on the relationship between soundscape indicators and attention restoration, without incorporating multisensory interactions (e.g., visual landscapes, olfactory perceptions, wind sensations, and tactile textures). Although soundscapes are a fundamental dimension of environmental experience (Kang et al., 2016), the quantification of “full-sensory experiences” (e.g., the pleasantness of bird songs may synergistically enhance visual perceptions of greenery and natural odors) remains to be further elucidated. Future research should integrate multimodal sensors to explore the impact of cross-sensory interactions on restorative experiences (Jo and Jeon, 2020). Second, this study utilized a GBDT model to establish a mapping relationship between soundscape indicators and soundscape descriptors (e.g., pleasantness, event richness). However, the study did not validate whether predicting soundscapes can produce the same attention recovery effects as actual environments. Currently, the study only extracts 479 feature indicators related to campus-specific object-level features, semantic-level features, scene types, and pixel-level features to predict campus soundscapes. However, acoustic indicators from the visual domain cannot fully capture the complex subjective auditory experience. This limitation manifests in three aspects (Zhong et al., 2025): first, the introduction of ecological acoustic indices (EIs) into traditional soundscape indicators (SIs) improved the predictive power of all eight soundscape descriptors. Second, EIs are more important in predicting eventfulness and vitality, with total relative importance ranging from 36.8% to 53.4%. Finally, indicators such as biological richness (BIO), olfactory intensity (Ht), event count (NP), and human disturbance level (NDSI) have a significant impact on at least one soundscape descriptor and are considered key EIs. Therefore, future research urgently needs to integrate semantic analysis methods, deep learning methods, and ecological acoustic indices (EIs) to achieve a more comprehensive understanding of subjective soundscape experiences and enhance their explanatory power.

## 5 Summary

This research, focusing on Fuzhou City University as a case study, proposed a framework integrating image-based soundscape prediction, cross-modal visual emotion analysis, and visual perception modeling to inform the strategic planning of campus audiovisual environments. The methodology involved gathering PRS-11 questionnaire data, performing street map crawling and prediction, conducting large-scale analysis of 114 student image evaluations using a CNN-BiLSTM-based framework, and applying machine learning techniques to model the interaction between campus audiovisual environment indicators and attention restoration quality. Additional analysis and visualization were carried out utilizing ArcGIS software. This study uncovered pivotal elements of the campus audiovisual environment that significantly impact attention restoration quality and outlined strategic recommendations for the sustainable improvement of the university setting. The key findings are summarized as follows:

(1) There are significant differences in the distribution of the quality of attention restoration between each university, and the quality of attention restoration in universities dominated by liberal arts and agricultural disciplines is significantly higher than that of schools in the science and technology category, and schools in the agriculture and forestry category have a boundary effect, which may be reflected in the transition between natural and artificial landscapes, for example, between fields and laboratories, or the boundaries between different agricultural areas (e.g., croplands, woodlands, etc.). These boundaries help to create a unique sense of “space” and “ecology” that provides a psychological buffer between academic and hands-on tasks, allowing students to regain focus and emotional balance in complex work environments. The significantly higher quality of attention recovery in liberal arts schools is partly due to their unique academic atmosphere, humanistic environment, and intertwined relationship with nature and culture, which provides a good platform for students to recover through various factors such as spatial design and social interaction.
(2) SHAP analysis results reveal that the most influential ecological factors, ranked by importance, include cultural soundscapes, natural acoustic elements, chromatic complexity, mechanical noise, visual sentiment, and aquatic features. Among them, humanistic sound is greater than −0.25, and more than 1.25 will have destabilizing effects, and natural sound should be controlled between −1 and 1. Color complexity alone is mainly clustered between −2 and 2, however, only between −2 and 0 can show a positive contribution. It is also vital to pay attention to water feature values, green space and enclosed space as they may have some effect on individuals.
(3) This research highlights the critical need to account for the non-linear impacts of environmental factors on individuals when planning and designing campus audiovisual settings. Greater enclosure levels can substantially amplify the beneficial effects of vegetation density, while the interaction between vegetation density and color complexity with paving coverage can further elevate positive emotional responses. Additionally, variations in paving materials and the extent of paved surfaces may influence individuals' perception and appreciation of soundscapes. Color complexity serves as a moderating factor for emotional states in human environments. High levels of musical acoustics, balanced visual entropy, and the proportion of waterscape, when combined, tend to generate predominantly adverse emotional outcomes.

In conclusion, when planning campus audiovisual landscapes for environmental sustainability, the design should focus on the characteristics of institutions in different disciplinary categories, where building infrastructure, audiovisual design environments are crucial. This research underscores the significance of accurate metrics within the campus audiovisual built environment in enhancing university students' attention restoration. It also addresses a critical gap in prior studies regarding the assessment of campus audiovisual environment indicators. These findings provide practical insights for assessing and designing campus audiovisual environments.

## Data Availability

The raw data supporting the conclusions of this article will be made available by the authors, without undue reservation.
