# Supplementary figures and images for: Exploring the relationship between audio–visual perception in Fuzhou universities and college students' attention restoration quality using machine learning
Source: Front Psychol. 2025 Jul 4;16:1572426. doi: 10.3389/fpsyg.2025.1572426 (PMC12271214; doi:10.3389/fpsyg.2025.1572426)

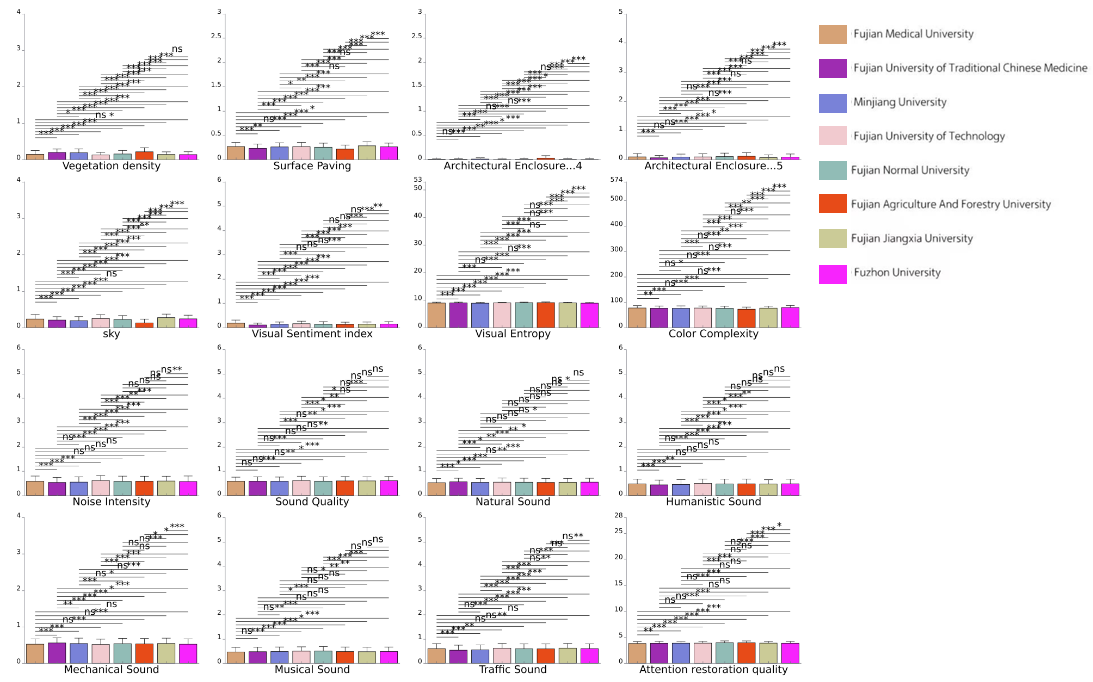

Supplement: Supplementary file 1 [file Data_Sheet_1.doc]
